# Remarkable Case of Fractured Skull, with the Appearances on Dissection

**Published:** 1817-04

**Authors:** 

**Affiliations:** Surgeon R. N.


### *Remarkable Case of Fractured Skull, with the Appearances on Dissection.*

By Mr. Morrison, Surgeon R. N.

August 13th, 1815. Francis Martin, aged 24, a stout muscular man, at six o'clock this morning fell from about the height of twenty feet, out of the main rigging on deck. The fall was somewhat interrupted by his striking against the gunwale of a boat on the booms. At first, the pulse was very slow, and hardly perceptible at the wrist. Th[illegible] patient perfectly insensible; pupils dilated, and insensi-

---

ble to the stimulus of candle-light. He alighted principally on his face and left side. On being stripped, I found the left radius fractured, about two inches from the carpal extremity. A few minutes after the accident, he voided considerable quantities of blood by the nose and mouth, and uttered heavy groans. At this time the pulse rose fast. The patient was bled immediately to about *seventy ounces* before the pulse sunk, or *deliquium animi* was induced. During the blood-letting sensibility returned, and he complained of much pain in the forehead and face, as well as in his left arm. His face was much bruised, and a crepitus, although obscure, was discovered to exist between the nasal and frontal bones; a desire to sleep succeeded the blood-letting, and he was put into bed, the fractured limb being secured by means of splints and bandages.

℞. Antim. tart. gr. iij, misce cum aquæ font. ℥viij; sumat ℥j secunda quâque horâ.

*Nine o'clock, p. m.* Soon after having taken the second portion of the medicine, he vomited at least two quarts of a dark grumous liquid mixed with coagulated blood; in about half an hour after, vomiting recurred, and still dark, but no blood.—*Ten o'clock, p. m.* Pulse 96, inclining to be sharp. Blood-letting repeated to sixteen ounces. Mixture continued.

*August 14th, four o'clock, a. m.* Before the vein was tied up last night, the pulse sunk considerably; and since that he has enjoyed about five hours sleep. At present, pulse 114, and sharp: says he feels free from all pain, save in the head and arm. A tumefaction pervades the whole of his face; and in consequence, he is unable to open either eye. Has taken the rest of the mixture, without producing any sensible change.—*Ten o'clock, a. m.* Continues restless; heat of skin above the natural standard. Pulse same as at four o'clock; tongue clean; no stool.

℞. Pulv. jalapæ, gr. xvj; calomel ppt. gr. xij, sum. stat. postea, Abrad. capill. tot. capit. Admoveat. emp. lyttæ part. super.

*August 15th, eight o'clock, a. m.* Has passed a tolerable night, and expresses himself as better; pulse 72, sharp; skin hot and dry; tongue moist and clean: has had one copious loose stool, mixed with coagulated blood. Œdema of face diminished. Repetat. venesectio, postea habeat pulv. jalapæ ʒß; supertart. potassæ ʒij.—*Five*

*o'clock, p. m.* Only ten ounces of blood could be taken away, when the pulse sunk. The blood, in this instance, exhibited no inflammatory appearance. No stool.

℞. Pulv. jalapæ gr. iv; calomel ppt. gr. iij; mucilag. acaciæ q. s. ut ft. pilulæ duæ, capiendæ secunda quâque horâ, donec alvus plene respond.

*August 16th, eight, a. m.*—Had a tolerable night's rest; no stool, after having taken ten of the pills. Pulse 76, weak; skin rather hot; tongue clean. Œdema of the face trifling; can now open both eyes. Blisters, after remaining thirty-six hours, produced but a slight serous discharge. Complains of his mouth being tender.

℞. Massæ pil. aloet. gr. xxx, statim sumend.—*Five o'clock, p. m.* No stool.—℞. Ol ricini ℥iß.

*August 17th, eight o'clock, a. m.* Last evening had one copious loose stool, soon after taking the castor oil. Afterwards had a good night's rest; and, this morning, feels himself pretty easy.

*August 18th.* Little or no alteration in general health since yesterday's report; has had two copious dark-coloured stools.

*August 19th.* Improves slowly; and although he is irritable occasionally, there being no active symptom now but the pain of forehead, which does not prevent natural rest, he may be looked upon in a fair way of recovery. Breath strongly impregnated with mercury. Sago and sugar for diet.

*August 20th.* Continues to rest well, but is irritable when any thing is denied him. Mercurial fœtor of the breath not so perceptible as yesterday. Tongue clean; skin dry; pain of head continues nearly uniform. A blister to the nape of the neck. Continue the solution of tartarized antimony.

*August 21st.* No perceptible change from yesterday; pulse 84, compressible and feeble. Tongue clean. No stool since the evening of the 19th. The blister is removed, without producing the desired effect, after remaining on 34 hours. To have rice and sugar. *Five o'clock, p. m.* Partook of the rice liberally; a few minutes afterwards had an indistinctly formed rigor, and continues since in a state of stupor; pulse 112, compressible, and fluttering: left eye protrudes much out of the socket; pupils dilated, and the left eye only sensible to candle-light. Breathing hurried and oppressive. Sinking fast! To have some white-wine negus. *Six oclock, p. m.* Pulse hardly perceptible at the wrist; subsultus tendinum. He gradually sunk, and expired at half past seven o'clock, without uttering a groan.

*Dissection, sixteen hours post mortem.* Upon laying bare the cranium, a fracture was observed to extend from the left os nasi upwards to the coronal suture; and another from the external canthus of the left eye, in the shape of an arch, joining with the former: from this arched fracture extended another short one, towards the ear of the same side. From the right zygomatic process another fracture extended upwards and backwards about three inches; neither of the fractures overlapped, or permitted the probe to enter. On removing the calvarium, two small coagula of blood adhered to the bone, above the right orbit. The *cerebrum seemed to be in a healthy state,* save a small portion of the anterior part of the left lobe, which was partly absorbed, and a thick brownish matter covering its surface, occasioned by numerous spiculæ of projecting bone. On removing the brain, the fractures presented terrible appearances; the bones composing the left orbit were shattered into several pieces, and pressing on the substance of the brain; the bones composing the right orbit were neither so much shattered nor displaced. The coats of the right eye were greatly absorbed away, and nearly ready to let the vitreous humour escape. The anterior portion of the sphenoid bone was displaced; and the zygoma was loose. Laying open the thorax, slight adhesions presented in the right cavity, evidently from previous disease. The liver seemed *very large,* but I could not detect any morbid structure. All the other viscera were sound.

*Observations.* The degree of inflammation in the brain here, presented a striking contrast with the degree of injury in its bony case. The length of time during which life was preserved, gave full space for the developement of inflammatory disorganization; and the *post mortem* appearances offer an instructive lesson on the power of copious blood-letting in cases of extensive cranial fractures. Had it not been for the spiculæ of bone that wounded the anterior lobe of the cerebrum, there is every probability that recovery would have taken place, notwithstanding the dreadful series of fractures which dissection disclosed. The enlarged state of the liver, was it connected with the cerebral disease? EDITORS.

